# Association between *Helicobacter pylori* Infection and Diabetes: A Cross-Sectional Study in China

**DOI:** 10.1155/2020/7201379

**Published:** 2020-09-24

**Authors:** Sailimai Man, Yuan Ma, Cheng Jin, Jun Lv, Mingkun Tong, Bo Wang, Liming Li, Yi Ning

**Affiliations:** ^1^Department of Epidemiology and Biostatistics, School of Public Health, Peking University Health Science Center, Beijing 100191, China; ^2^Meinian Institute of Health, Beijing 100191, China; ^3^Peking University Health Science Center Meinian Public Health Institute, Beijing 100191, China

## Abstract

**Background:**

Studies suggest an association between *H. pylori* infection and extragastrointestinal disease. Limited studies provided conflicting results on the association between *H. pylori* infection and diabetes. The present study was aimed at examining the association between *H. pylori* infection and diabetes in a large health checkup population in China.

**Methods:**

A cross-sectional study was conducted; participants who attended health checkups at Beijing MJ Health Screening Center during 2017-2018 were included. *H. pylori* infection was diagnosed by ^13^C-urea breath test. Multivariate logistic regression analysis was performed to evaluate the association between *H. pylori* infection and diabetes.

**Results:**

The mean age of 13,397 participants was 43.8 ± 12 years. The prevalence of *H. pylori* infection and diabetes was 28.2% and 8.1%, respectively. The prevalence of diabetes was higher among *H. pylori*-positive participants compared with their counterparts (8.9% vs 7.8%, *p* = 0.05). After adjustment of age, sex, family history of diabetes, smoking, education, stroke, coronary heart disease, BMI, SBP, TG, HDL-C, and LDL-C, multivariate logistic regression analysis found no association between H. pylori infection and diabetes (OR 1.02, 95% CI 0.88-1.18). Additionally, subgroup analysis indicated that *H. pylori* infection was significantly associated with increased risk of diabetes in the female group (OR 1.09, 95% CI 1.08-1.09).

**Conclusions:**

No significant association was found between *H. pylori* infection and diabetes. However, the subgroup analysis suggested that *H. pylori* infection was possibly associated with increased risk of diabetes among females. Future cohort studies are needed to verify this association in females and to address possible implication in the prevention of diabetes.

## 1. Introduction

Diabetes has become a major public health problem with rapidly increasing prevalence worldwide. According to the International Diabetes Federation, 425 million people worldwide were estimated to have diabetes in 2017 [1]. A recent national survey revealed that the prevalence of diabetes in China was 10.9% in 2013 [2]. It is worthy of note that 114 million people in China were estimated to have diabetes in 2017, which means China has the world's largest diabetes epidemic [3]. Under this circumstance, identification of specific risk factors of diabetes in China will be of particular significance.

*Helicobacter pylori* (*H. pylori*) infection has been identified as a public health threat worldwide; approximately 50% of the world population is estimated to be infected with *H. pylori* [4]. Its prevalence has been demonstrated to range considerably according to socioeconomic status and tends to be more serious in developing countries [4, 5]. In China, the prevalence of *H. pylori* infection ranges from 28.0% to 73.3% in different regions of the country [6, 7]. *H. pylori* can commonly lead to gastrointestinal complications, including chronic gastritis, peptic ulcer, gastric adenocarcinoma, and mucosa-associated lymphoid tissue lymphoma [8, 9]. Notably, numerous studies have revealed significant association between *H. pylori* infection and extragastrointestinal conditions, such as cardiovascular disease, autoimmune disease, neurological disease, hyperemesis gravidarum during pregnancy, and metabolic disorders [10–14]. In particular, *H. pylori* infection is suspected to be associated with diabetes, which has become an intriguing field in recent years [15, 16].

However, existing studies addressing the association between *H. pylori* infection and diabetes provided conflicting results [17–21]. If *H. pylori* infection contributes to the incidence of diabetes, eradication of *H. pylori* infection may play an important role in diabetes prevention and control. Therefore, the objective of the current study was to examine the association between *H. pylori* infection and diabetes in a large medical checkup population in China.

## 2. Methods

### 2.1. Study Population

This was a cross-sectional study, using existing health checkup data from participants who attended routine annual health checkups at Beijing MJ Health Screening Center (Beijing, China) during 2017-2018. The health checkup data included anthropometric parameters, serum biochemical indices, and ^13^C-urea breath test. Meanwhile, demographic characteristics, self-reported lifestyle, and medical conditions were collected by a questionnaire during the health checkup. We excluded subjects aged <18 years and those with missing data on *H. pylori* infection, diabetes, family history of diabetes, hypertension, smoking, BMI, systolic blood pressure (SBP), diastolic blood pressure (DBP), total cholesterol (TC), triglyceride (TG), high-density lipoprotein cholesterol (HDL-C), low-density lipoprotein cholesterol (LDL-C), FPG, or HbA1c. This study has been approved by the Institutional Review Board of Peking University Health Science Center (ID of the approval: IRB00001052-19077). Individual informed consent was waived, as only anonymized data were used in this study.

### 2.2. Diagnosis of Diabetes

In the current study, diabetes was defined according to the American Diabetes Association criteria as meeting any of the following criteria [22]: (i) FPG ≥ 7.0 mmol/L; (ii) HbA1c ≥ 6.5%; and (iii) self-reported physician diagnosis of diabetes or use of antidiabetic medication. Previous studies have confirmed that self-reported diabetes is a valid method to evaluate people's diabetes status in a Chinese and other population [23–25]. Fasting plasma glucose (FPG) was measured using the hexokinase method (Cobas 8000 modular analyzer, Roche Diagnostics). HbA1c was assayed using the ion-exchange high-performance liquid chromatography method (G8 HPLC Analyzer, Tosoh).

### 2.3. Establishment of *H. pylori* Infection

The ^13^C-urea breath test was applied to diagnose *H. pylori* infection. After overnight fasting for at least 8 h, participants were required to take 75 mg of ^13^C-urea (Urea-^13^C Capsule Breath Test Kit, HEADWAY) after providing the initial baseline breath sample. We collected the second breath sample after half an hour. The samples were detected using a ^13^C-urea breath test analyzer (HCBT-01, HEADWAY). *H. pylori* infection was defined as positive if the difference between the two samples exceeded 4.0 parts per 1,000 of ^13^CO_2_. Otherwise, it was considered negative.

### 2.4. Assessment of Covariates

Demographic characteristics (age, sex), lifestyle, and medical conditions were collected by a self-reported questionnaire. Level of education was divided into two categories according to whether individual participant received a college education (education time ≤ 12 or >12 years). Smoking was classified as never smoker, former smoker, and current smoker. Family history of diabetes, stroke, and coronary heart disease were divided into two categories as yes or no. Standard laboratory methods were applied to obtain TC, TG, LDL-C, and HDL-C. Physical examinations, including height, weight, and blood pressure (BP), were performed by trained physicians. BMI was calculated as weight in kilograms divided by height squared in meters. BP was measured with a regular mercury sphygmomanometer on the right arm while seated after resting for at least 5 min. Hypertension was defined as meeting any of the following criteria: (i) systolic blood pressure (SBP) ≥ 140 mmHg; (ii) diastolic blood pressure (DBP) ≥ 90 mmHg; and (iii) self-reported physician diagnosis of hypertension or use of antihypertensive medication.

### 2.5. Statistical Analysis

Characteristics of the participants are described as mean ± SD for continuous variables with a normal distribution, median (interquartile range, IQR) for continuous variables with a skewed distribution, and percentages for categorical variables. Characteristics of participants across *H. pylori* status were compared using Student's *t*-test, Wilcoxon rank test, or *χ*^2^ test. To examine the association between *H. pylori* infection and diabetes, multivariate logistic regression analysis was performed to estimate adjusted odds ratios (ORs) and 95% confidence intervals (CIs). Age- and sex- adjusted model (model 1) was performed first. Then, family history of diabetes (yes or no), smoking status (never smoker, former smoker, and current smoker), and level of education (≤12 or >12 years) were further added into model 2. Finally, we added potential risk factors of diabetes and confounders, including stroke (yes or no), coronary heart disease (yes or no), BMI (continuous variable), SBP (per 5 mmHg, continuous variable), TG (continuous variable), HDL-C (continuous variable), and LDL-C (continuous variable) into model 3, to further evaluate the association between *H. pylori* infection and diabetes.

Subgroup analysis was performed using characteristics including age (<60 or ≥60 years old), sex (male or female), family history of diabetes (yes or no), hypertension (yes or no), smoking status (never smoker, former smoker, and current smoker), level of education (≤12 or >12 years), BMI (<24 or ≥24 kg/m^2^), TG (<1.7 or ≥1.7 mmol/L), HDL-C (<1.0 or ≥1.0 mmol/L), and LDL-C (≤4.1 or >4.1 mmol/L) in the fully adjusted model (model 3). Alongside the grouping variable, other variables were used as adjustment variables. Statistical analysis was conducted by SAS 9.2 (SAS Institute, Cary, NC, USA). Two-tailed *p* values < 0.05 were considered statistically significant.

## 3. Results

A total of 13,397 participants (5,978 female and 7,419 male) were included in this study. The mean age of the participants was 43.8 ± 12 years. The prevalence of *H. pylori* infection and diabetes was 28.2% and 8.1%, respectively. The prevalence of diabetes was higher among *H. pylori*-positive participants compared to their counterparts (8.9% vs. 7.8%, *p* = 0.05).  Table 1 presented the characteristics of the participants across *H. pylori* status. Among all the participants, 18.1% have a family history of diabetes. Participants who had *H. pylori* infection were more likely to be males, smokers, and hypertensive with lower educational level and also had higher levels of BMI, SBP, DBP, TG, TC, LDL-C, FPG, and HbA1c, but lower level of HDL-C.

### 3.1. Associations between *H. pylori* Infection and Diabetes

Table 2 displayed the results of different models in the multivariate logistic regression analysis. The unadjusted OR for the association between *H. pylori* infection and diabetes was 1.15 (95% CI 1.00-1.31; *p* = 0.05). After adjustment for age and sex, *H. pylori* infection was associated with an increased risk of diabetes (OR 1.14, 95% CI 0.99-1.32) but without statistical significance (*p* = 0.07). Additional adjustment for family history of diabetes, smoking, and education obtained attenuated the result (OR 1.10, 95% CI 0.95-1.28; *p* = 0.19). In the fully adjusted analysis, *H. pylori* infection was not significantly associated with diabetes (OR 1.02, 95% CI 0.88-1.18; *p* = 0.81), after further adjustment for stroke, coronary heart disease, BMI, SBP, TG, HDL-C, and LDL-C.

### 3.2. Subgroup Analysis for the Association between *H. pylori* Infection and Diabetes

The association of *H. pylori* infection with diabetes was investigated according to different subgroups, including age, sex, family history of diabetes, hypertension, smoking, education, BMI, TG, HDL-C, and LDL-C (Table 3). *H. pylori* infection was significantly associated with diabetes among females (OR 1.09, 95% CI 1.08-1.09; *p* < 0.001). For all the other subgroups, no significant associations were found between *H. pylori* infection and diabetes (all *p* > 0.05).

## 4. Discussion

In the present study, we found no significant association between *H. pylori* infection and prevalent diabetes after adjustment for risk factors of diabetes and potential confounders. However, subgroup analysis did find an association between *H. pylori* infection and increased risk of prevalent diabetes among females.

The relationship between *H. pylori* infection and diabetes remained controversial for the last years. Several studies supported our results on the lack of association between *H. pylori* infection and diabetes [17, 20, 26]. In contrast, others found *H. pylori* infection to be positively associated with diabetes [19, 21, 27, 28]. A meta-analysis that included 41 case-control studies concluded that *H. pylori* infection could be a potential risk factor for type 2 diabetes, while among them, two studies conducted in China both showed a negative result [29]. Nevertheless, few of these previous studies had adjusted family history of diabetes in their analysis. Since there has been compelling evidence supporting the strong role of genetics in the development of diabetes [30, 31], the relationship between *H. pylori* infection and diabetes cannot be truly clarified without adjusting family history of diabetes.

Although there is no strong evidence about *H. pylori* infection resulting in diabetes and the underlying mechanism is not clear, it is biologically plausible. Some researchers believed that chronic inflammation and insulin resistance induced by *H. pylori* may increase the risk for diabetes [32, 33]. *H. pylori* colonization can trigger inflammation responses. Neutrophil cells penetrate the gastrointestinal mucus at the initial phases of infection. Then, at the chronic phase of infection, those cells are replaced by monocyte instead. Monocyte is capable of producing a variety of inflammatory cytokines (mainly including tumor necrosis factor-*α*, interleukin, and C-reactive protein) that, in addition to exerting local effects, can also be released on other tissues and organs and ultimately cause an increase in extragastrointestinal conditions [34, 35]. Those inflammatory cytokines probably contribute to diabetes by causing insulin resistance and are in turn intensified in the presence of hyperglycemia to promote long-term complications of diabetes [36]. Several studies considered these inflammatory cytokines as the connection between *H. pylori* and diabetes [37, 38], and some found the connection with sex difference [39, 40], while others found that the connection adds little to the association between *H. pylori* and diabetes once classical risk factors for diabetes have been accounted for [41, 42].

More studies are necessary to elucidate the pathogenesis of *H. pylori* resulting in diabetes. It is worth to mention that results of some studies have indicated that *H. pylori* cytotoxin-associated gene A (CagA), which has been identified as a possible virulence marker of *H. pylori* [43], induces more severe inflammatory responses [44]. Future studies should examine the effect of CagA positivity in the process of *H. pylori*-induced diabetes. Besides, the duration of *H. pylori* infection is also worth of concern. Compared to subjects with short-term infection, those with more longstanding infection may have more severe mucosal damage and inflammatory responses and thereby develop insulin resistance.

Notably, growing evidence showed that inflammation process and insulin resistance were highly depending on sex and sex hormone status throughout the lifetime [45–47]. A study had identified a direct interaction between sex hormones and microbial exposures and showed that microbiome manipulations can provoke a testosterone-dependent effect on inflammation and autoimmunity in a genetically high-risk rodent model. The transfer of gut microbiota from adult males to immature females altered the recipient's microbiota and testosterone level, resulting in metabolomic changes, reduced islet inflammation and autoantibody production, and ultimately robust type 1 diabetes protection [48, 49]. In the present study, we found that *H. pylori* infection was associated with increased risk of diabetes only among females. Based on the evidence provided by previous studies, we speculated that the sex discrepancy of diabetes susceptibility found in our study was likely due to the interaction between sex hormones and *H. pylori*-induced inflammation.

Increased inflammation induced by *H. pylori* infection could lead to insulin resistance and diabetes through a sex-dimorphic hypothalamic insulin action. Studies using a rat model have proved that inflammation was a potential cause for impaired insulin action within the hypothalamus [50]. Specifically, systemic inflammation induced by *H. pylori* infection could increase the expression of proinflammatory cytokines such as interleukin-6 and tumor necrosis factor-*α*. Those cytokines were able to act on the hypothalamus and result in hypothalamic inflammation [51]. Hypothalamic inflammation could reduce insulin signaling and was an important cause of the disrupted neuroendocrine control of metabolism that resulted in systemic insulin resistance [52, 53], whereas insulin signaling and action were found to be more disrupted in female rats than in male rats [50]. The underlying mechanism was the modulation caused by brain estrogen signaling, potentially mediated by estrogen effects on ER*α* in various regions of the hypothalamus [54, 55]. The fundamental sex difference in central insulin signaling was not only found in animals but also corroborated in humans [54, 56]. The joint effect of *H. pylori*-induced inflammation and sex hormones mentioned above might explain for the more pronounced insulin resistance among females and finally made them more predisposed to diabetes compared with males.

The present study had several strengths. First, we fully adjusted potential confounders and risk factors of diabetes in the multivariate analysis, which made the results more reliable and convincing. Few of the previous studies, especially those that found a significant association between *H. pylori* and diabetes, included family history of diabetes into their multivariate regression models. This probably biased the results. Second, the diagnosis of *H. pylori* infection in our study was established by the ^13^C-urea breath test rather than serologic testing. Previous studies have found that there is a need for an optimization of ^13^C-urea breath test threshold levels, while the ^13^C-urea breath test is still believed to be the most widely available and accurate noninvasive test for *H. pylori* diagnosis compared with serologic testing, which was commonly used in previous studies [57, 58]. Despite the strengths, there were still some limitations in our study. First, due to the cross-sectional design, causal relationship between *H. pylori* infection and diabetes could not be determined. Larger-scale prospective cohort studies are required to investigate the association between *H. pylori* infection and risk of incident diabetes. Second, possible confounding factors, i.e., polycystic ovary syndrome, total energy intake, and physical activity, were not adjusted in the regression analysis, as they were not precisely measured in the health checkup population. However, we adjusted BMI in our analysis, which to some extent could represent the effect of total energy intake and physical activity. Granted the possible confounding factors and the small effect sizes we calculated, the observed association between *H. pylori* infection and diabetes among females in the present study may be statistically but not clinically significant [59]. Only after critically examining the clinical significance and relevance of this association should it be applied to clinical situations.

## 5. Conclusions

In summary, we found no significant association between *H. pylori* infection and diabetes. However, an increased risk of prevalent diabetes was found among females with *H. pylori* infection. Large-scale, well-designed prospective studies are needed to investigate if *H. pylori* plays an etiological role in the development of diabetes.

## Figures and Tables

**Table 1:** Characteristics of participants according to the status of *H. pylori* infection^†^.

Characteristics	*H. pylori* negative (*N* = 9,622)	*H. pylori* positive (*N* = 3,775)	*p* value
Age (years)	43.8 ± 12.2	43.8 ± 11.9	0.96
Male, *n* (%)	5,168 (53.7)	2,251 (59.6)	<0.001
Diabetes, *n* (%)	753 (7.83)	335 (8.87)	0.05
Family history of diabetes, *n* (%)	1,787 (18.6)	641 (17.0)	0.03
Hypertension, *n* (%)	1,571 (16.3)	720 (19.1)	<0.001
Smoking status, *n* (%)			<0.001
Never smoker	7,483 (77.8)	2,740 (72.6)	
Former smoker	433 (4.50)	178 (4.72)	
Current smoker	1,706 (17.7)	857 (22.7)	
Level of education, *n* (%)			<0.001
≤12 years	459 (4.77)	279 (7.39)	
>12 years	5,710 (59.3)	2,070 (54.8)	
Missing	3,453 (35.9)	1,426 (37.8)	
BMI (kg/m^2^)	23.9 ± 3.44	24.5 ± 3.50	<0.001
SBP (mmHg)	117 ± 14.8	118 ± 15.3	<0.001
DBP (mmHg)	72.1 ± 10.8	73.1 ± 11.1	<0.001
TC (mmol/L)	4.63 ± 0.89	4.68 ± 0.90	0.004
TG (mmol/L)	1.39 ± 1.03	1.48 ± 1.12	<0.001
HDL-C (mmol/L)	1.43 ± 0.40	1.38 ± 0.38	<0.001
LDL-C (mmol/L)	3.04 ± 0.81	3.10 ± 0.82	<0.001
FPG (mmol/L)	5.62 ± 1.00	5.71 ± 1.16	<0.001
HbA1c (%)	5.60 ± 0.61	5.64 ± 0.71	0.008

^†^Characteristics of participants across *H. pylori* status were compared using Student's *t*-test, Wilcoxon rank test, or *χ*^2^ test. BMI: body mass index; SBP: systolic blood pressure; DBP: diastolic blood pressure; TC: total cholesterol; TG: triglyceride; HDL-C: high-density lipoprotein cholesterol; LDL-C: low-density lipoprotein cholesterol; FPG: fasting plasma glucose. Data are mean ± standard deviation or *n* (%).

**Table 2:** Association between *H. pylori* infection and diabetes.

Model^†^	*H. pylori* negative	*H. pylori* positive	OR (95% CI)	*p* value
Unadjusted model	753 (7.83)	335 (8.87)	1.15 (1.00-1.31)	0.05
Model 1	753 (7.83)	335 (8.87)	1.14 (0.99-1.32)	0.07
Model 2	753 (7.83)	335 (8.87)	1.10 (0.95-1.28)	0.19
Model 3	753 (7.83)	335 (8.87)	1.02 (0.88-1.18)	0.81

^†^Model 1: adjusted for age and sex. Model 2: model 1 + family history of diabetes, smoking status, and level of education. Model 3: model 2 + stroke, coronary heart disease, BMI, SBP, TG, HDL-C, and LDL-C. Data are *n* (%).

**Table 3:** Subgroup analysis of the association between *H. pylori* infection and diabetes^†^.

Subgroup	n	*H. pylori* negative	*H. pylori* positive	OR (95% CI)	*p* value
All	13,397	9,622 (71.8)	3,775 (28.2)	1.02 (0.88-1.18)	0.81
Age (years)					
<60	11,941	482 (5.63)	226 (6.70)	1.00 (0.84-1.20)	0.97
≥60	1,456	271 (25.7)	109 (27.1)	1.01 (0.76-1.34)	0.93
Sex					
Male	7,419	538 (10.4)	245 (10.9)	1.00 (0.84-1.20)	0.97
Female	5,978	215 (4.83)	90 (5.91)	1.09 (1.08-1.09)	<0.001
Family history of diabetes					
Yes	2,428	224 (12.5)	94 (14.7)	1.03 (0.76-1.39)	0.80
No	10,969	529 (6.75)	241 (7.69)	1.02 (0.86-1.22)	0.87
Hypertension					
Yes	2291	339 (21.6)	166 (23.1)	1.16 (0.92-1.46)	0.52
No	11,106	414 (5.14)	169 (5.53)	0.94 (0.77-1.14)	0.21
Smoking status					
Never smoker	10,223	457 (6.11)	189 (6.90)	1.03 (0.84-1.25)	0.80
Former smoker	661	68 (15.7)	27 (15.2)	1.02 (0.59-1.74)	0.96
Current smoker	2,563	228 (13.4)	119 (13.9)	1.03 (0.79-1.35)	0.81
Level of education					
≤12 years	738	99 (21.6)	57 (20.4)	0.88 (0.58-1.33)	0.54
>12 years	7,780	374 (6.55)	149 (7.20)	1.05 (0.85-1.31)	0.64
Missing	4,879	280 (8.11)	129 (9.05)	1.00 (0.78-1.27)	0.97
BMI (kg/m^2^)					
<24	6,926	215 (4.14)	75 (4.34)	1.10 (0.82-1.47)	0.54
≥24	6,471	538 (12.2)	260 (12.7)	1.00 (0.84-1.18)	0.96
TG (mmol/L)					
<1.7	10,040	418 (5.70)	156 (5.75)	0.91 (0.74-1.12)	0.40
≥1.7	3,357	335 (14.6)	179 (16.9)	1.16 (0.93-1.44)	0.18
HDL-C (mmol/L)					
<1.0	1,822	182 (14.8)	80 (13.6)	0.86 (0.63-1.18)	0.35
≥1.0	11,575	571 (6.81)	255 (8.00)	1.08 (0.91-1.28)	0.38
LDL-C (mmol/L)					
≤4.1	12,115	659 (7.55)	283 (8.36)	0.99 (0.84-1.16)	0.87
>4.1	1,282	94 (10.5)	52 (13.3)	1.16 (0.78-1.71)	0.47

^†^All the subgroup analyses were adjusted for age, sex, family history of diabetes, hypertension, smoking status, level of education, BMI, TG, HDL-C, and LDL-C. BMI: body mass index; TG: triglyceride; HDL-C: high-density lipoprotein cholesterol; LDL-C: low-density lipoprotein cholesterol. Data are *n* (%).

## Data Availability

All data used to support the findings of this study may be released upon application to the Meinian Institute of Health (Beijing, China), which can be contacted through Prof. Yi Ning (email: yi.ning@meinianresearch.com).
